# Agenesis of the piriformis muscle: A case report with review of literature

**DOI:** 10.4102/sajr.v24i1.1838

**Published:** 2020-04-16

**Authors:** Pankaj Nepal, Syed I. Alam, Sadia Sajid, Syeda S. Intakhab, Vijayanadh Ojili

**Affiliations:** 1 Department of Radiology, St. Vincent’s Medical Center, Bridgeport, United States; 2 Department of Clinical Imaging, Hamad Medical Corporation, Doha, Qatar; 3 Jawaharlal Institute of Postgraduate Medical Education and Research (JIPMER), Puducherry, India; 4 Department of Radiology, University of Texas Health, San Antonio, United States

**Keywords:** piriformis muscle, agenesis, MRI, orthopaedics, lower back pain

## Abstract

Agenesis of the piriformis muscle is an extremely rare occurrence. Knowledge about this anatomic variant is important because of its close proximity with the sciatic nerve and sacral plexus. The piriformis muscle also serves as an important anatomic landmark for image-guided intervention and hip surgery. We report a case of piriformis muscle agenesis in a 28-year-old woman, incidentally detected on magnetic resonance imaging of the lumbosacral spine and pelvis, performed for low back pain.

## Introduction

The piriformis muscle is a pear-shaped structure located in the deep pelvis that functions as one of the six short external rotators of the hip, as well as the proximal abductor of thigh during hip flexion.^1^ The most accepted classification of piriformis variation by Beaton and Anson has described several variations of this muscle, but not agenesis.^1,2,3^ Piriformis agenesis is extremely rare, and thus the associated symptoms, clinical findings and therapeutic options remain elusive. By far, the importance of the piriformis muscle is related to its close proximity to the sacral plexus which is anterior to the muscle and the sciatic nerve, intimately positioned at the sciatic notch.^4^

## Case report

A 28-year-old woman presented to her primary care physician with progressive worsening of intermittent low back pain radiating into both legs over a 1-month period. The pain was described as a dull ache, aggravated by activity and relieved by rest. She also experienced perineal discomfort. Physical examination was unremarkable, with no neurological compromise. The range of motion of the lower lumbar spine was limited by pain and the hip movements were normal. Basic laboratory examinations were unremarkable.

**FIGURE 1:**
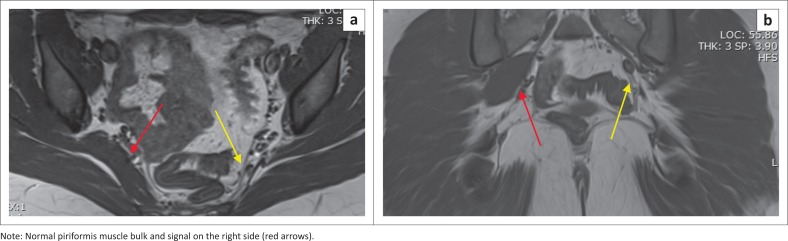
A 28-year-old woman who presented with low back pain for a month without neurological symptoms: (a) axial and (b) coronal T1-weighted images of the pelvis show congenital absence of the left piriformis muscle (yellow arrows point to the expected location).

**FIGURE 2:**
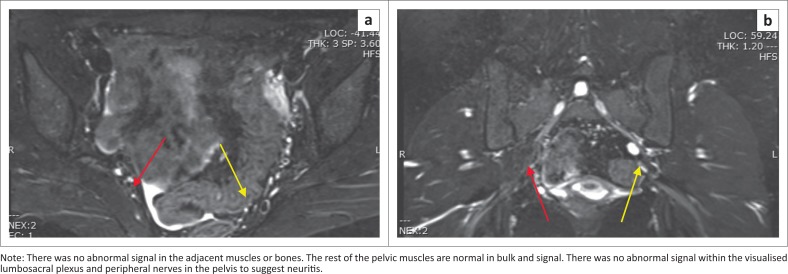
(a) Axial T2 fat-saturated (b) and coronal short tau inversion recovery images again reveal the same findings.

An initial radiograph of the lumbar spine was normal and a lumbosacral magnetic resonance imaging (MRI) was performed. The imaging findings were negative for disc disease, radiculopathy or cord abnormalities. The visualised skeleton and bilateral sacroiliac joints were also normal. Given the history of perineal discomfort, MR neurography of the pelvis was subsequently performed to evaluate the pudendal nerve and demonstrated absence of the left piriformis muscle (Figure 1). The sciatic nerve and lumbosacral plexuses were normal in configuration and signal intensity with no signs of neuritis (Figure 2). The patient was referred for physiotherapy along with a short course of analgesics. Her symptoms improved and she recovered completely at her 2-week follow-up assessment.

## Discussion

Although piriformis agenesis is rare, a recent meta-analysis in 2017 by Smoll et al.,^3^ suggested that other anatomic variations related to piriformis and the sciatic nerve complex are common, seen approximately in 17% of cadavers.^5^ To understand the congenital variants of the piriformis muscles, it is prudent to understand the basic anatomy. Piriformis originates from multiple deep pelvic structures, including the anterior surface of the lateral process of the sacrum, the gluteal surface of the ilium, the gluteal muscles and the capsule of the sacroiliac joint. It then passes through the greater sciatic notch, where the muscle is intimately related to the sciatic nerve. The tendon of the piriformis merges with tendons of the obturator internus and the superior and inferior gemilli to form a conjoint tendon that eventually inserts onto the greater trochanter of the femur.^1,2^

Different anatomic variants of the piriformis may exist. The most common variations are related to the anatomic relationship with the sciatic nerve.^4^ An accessory piriformis can exist, which may present as piriformis syndrome because of entrapment of the sciatic nerve at the sciatic notch.^4,6^ Other anatomic variants include a bifid piriformis and fusion with the adjacent gluteal, obturator or gemilli muscles. Clinical presentation of such variants is unknown. Following an extensive literature review, only a handful of case reports on the absence or agenesis of the piriformis muscle are documented.^5,7,8^ Only two cases have been reported in living human subjects.^5,8^

Because of the rarity in incidence, not much is known about the presenting symptoms, clinical findings and sequelae of piriform agenesis. None of the cases reported in the literature were suspected clinically but were discovered incidentally during imaging or cadaveric dissection. Abduction of the hip during walking is an important function not to overlook, as it prevents falling by shifting the body weight to the contralateral side.^1^ It is unknown whether this disturbed biomechanics influences stress on the spine and pelvis. Caetano et al.^5^ presented a case with neurologic symptoms of progressively worsening left buttock pain radiating to the left leg and localised tenderness of the ipsilateral ischiogluteal bursa on deep palpation; their patient demonstrated piriformis agenesis on the same side. However, Ikidag^8^ presented a case with left flank pain but with contralateral piriformis agenesis. Brenner et al.^7^ reported in a cadaveric dissection case, piriformis agenesis, an ipsilateral common gluteal artery and an absent inferior gemellus muscle. In our case, the patient had progressively worsening low back pain radiating to both legs without neurological deficits on examination. She also had perineal pain which improved after treatment of symptomatic pelvic inflammatory disease.

Because of the limited presentations, it remains elusive whether piriformis agenesis is clinically relevant. There is no definite data about the incidence of neurovascular complications during surgery or image-guided interventions associated with piriformis agenesis or its variations.^3^ Definitely, the piriformis is considered as an important landmark for image-guided interventions such as superior gluteal nerve block and sacral plexus block. It is also an important reference for the surgeon performing tendon release of various pelvic muscles and the posterior surgical approach in total hip arthroplasty.^7^ It can be postulated that the variation in the anatomy of such an important landmark definitely increases the risk of complications if the surgeon or interventionist is not aware.

Differential diagnoses for piriformis muscle agenesis may be congenital fusion with overlapping muscles, most commonly the superior gemellus and gluteus medius muscles, which can be differentiated with MRI. Other differentials include muscle involution or atrophy after injection of Botulinum toxin, iatrogenic damage after tenotomy or accidental rupture during total hip arthroplasty.^5^ Typical MR imaging features of chronic muscle tear are focal tendon thickening and peritendinous muscle atrophy which is not seen with muscle agenesis. Fatty involution after chronic muscle tear is seen characteristically as hyperintense on T1-weighted images. In doubtful cases, relevant clinical history of trauma or injury from intervention can rule out the same.

## Conclusion

Agenesis of the piriformis muscle is extremely rare. It is necessary for the radiologist and surgeon to be aware about this anatomical finding to avoid neurovascular mishaps whilst performing surgery or image-guided intervention. Because of the limited number of cases presented, it remains elusive whether it is an incidental finding on imaging or whether there may be altered biomechanics related to this variant.
